# Enhancing osteoporosis treatment with engineered mesenchymal stem cell-derived extracellular vesicles: mechanisms and advances

**DOI:** 10.1038/s41419-024-06508-w

**Published:** 2024-02-08

**Authors:** Yiman Chen, Yuling Huang, Jia Li, Taiwei Jiao, Lina Yang

**Affiliations:** 1 Departments of Geriatrics, The First Hospital of China Medical University, Shenyang, 110001 Liaoning PR China; 2 Department of Gastroenterology and Endoscopy, The First Hospital of China Medical University, Shenyang, 110001 Liaoning PR China; 3 Department of International Physical Examination Center, The First Hospital of China Medical University, Shenyang, 110001 Liaoning PR China

**Keywords:** Osteoporosis, Mesenchymal stem cells

## Abstract

As societal aging intensifies, the incidence of osteoporosis (OP) continually rises. OP is a skeletal disorder characterized by reduced bone mass, deteriorated bone tissue microstructure, and consequently increased bone fragility and fracture susceptibility, typically evaluated using bone mineral density (BMD) and T-score. Not only does OP diminish patients’ quality of life, but it also imposes a substantial economic burden on society. Conventional pharmacological treatments yield limited efficacy and severe adverse reactions. In contemporary academic discourse, mesenchymal stem cells (MSCs) derived extracellular vesicles (EVs) have surfaced as auspicious novel therapeutic modalities for OP. EVs can convey information through the cargo they carry and have been demonstrated to be a crucial medium for intercellular communication, playing a significant role in maintaining the homeostasis of the bone microenvironment. Furthermore, various research findings provide evidence that engineered strategies can enhance the therapeutic effects of EVs in OP treatment. While numerous reviews have explored the progress and potential of EVs in treating degenerative bone diseases, research on using EVs to address OP remains in the early stages of basic experimentation. This paper reviews advancements in utilizing MSCs and their derived EVs for OP treatment. It systematically examines the most extensively researched MSC-derived EVs for treating OP, delving not only into the molecular mechanisms of EV-based OP therapy but also conducting a comparative analysis of the strengths and limitations of EVs sourced from various cell origins. Additionally, the paper emphasizes the technical and engineering strategies necessary for leveraging EVs in OP treatment, offering insights and recommendations for future research endeavors.

## Facts

- Mesenchymal stem cells derived extracellular vesicles (EVs) have become a new direction for the treatment of degenerative orthopedic diseases.
- The information transmission function of EVs also makes them a key part in cell interactions.
- Engineering processing has been proven to improve the therapeutic effect of EVs.
- The development of microfluidic technology, 3D-bioprinting, and other technologies provides a possibility for large-scale extraction of EVs, which can be indispensable for clinical application.

## Open questions

- How do transplanted mesenchymal stem cells derived EVs influence crosstalk within the bone microenvironment?
- How can various engineering strategies be seamlessly integrated to maximize the efficiency of EV-based therapy?
- Are current EVs products suitable for conducting clinical trials?

## Introduction

Osteoporosis (OP) has emerged as an undeniable global public health concern. According to the International Osteoporosis Foundation (IOF), the number of high-risk individuals aged 50 and above for osteoporotic fractures worldwide was 158 million in 2010, expected to double by 2040 [1]. The economic burden of OP is immense; in the United States alone, the annual cost of osteoporosis-related fractures in 2005 was estimated at $17 billion, projected to rise to $25.3 billion by 2025 [2]. Functional impacts of osteoporosis-associated fractures include pain, dependency, depression, skeletal deformities, and impediments to essential daily activities. Fractures related to OP in the hip, vertebrae, and pelvis are common causes of morbidity and mortality in the elderly [3]. Numerous risk factors precipitate OP, such as aging, reduced mechanical stimulation, hormonal imbalance [4], and detrimental lifestyle habits [5], among others. These factors can potentially disrupt the dynamic equilibrium between osteoblast-mediated bone formation and osteoclast-driven bone resorption [6], imbalances in MSCs osteogenic and adipogenic differentiation [7], as well as oxidative stress-induced cellular DNA damage, apoptosis, and senescence [8]. Presently, pharmaceutical interventions predominate in the clinical treatment of OP [9]. Pharmaceuticals for OP can be primarily classified into two categories: 1. Anti-resorptive agents, such as bisphosphonates, denosumab, estrogens, and selective estrogen receptor modulators. 2. Anabolic medications, including teriparatide, a parathyroid hormone analog, which mitigates the risk of vertebral and non-vertebral fractures. The majority of these medications demonstrate a slow onset of action, and prolonged use may engender adverse events. For instance, long-term administration of anti-resorptive drugs may result in excessive bone hardening, compromising bone strength and flexibility, and augmenting the risk of non-compressive fractures [10]. Abaloparatide and teriparatide may elevate the risk of withdrawal due to adverse reactions (WAEs). Sustained bisphosphonate use may increase the risk of atypical femoral fractures (AFF) and osteonecrosis of the jaw (ONJ) [11]. In contemporary years, MSCs have emerged as a rapidly expanding area of investigation within the realm of regenerative medicine. Their application has yielded encouraging safety and efficacy profiles in the clinical management of a diverse array of pathologies, encompassing graft-versus-host disease, traumatic spinal cord lesions, autoimmune disorders, as well as skeletal and cartilaginous injuries [12]. Specifically in the context of bone and cartilage damage-associated disorders, a plethora of research has corroborated the therapeutic promise of MSCs for addressing orthopedic ailments via preclinical experiments and clinical inquiries [13].

**Fig. 1:**
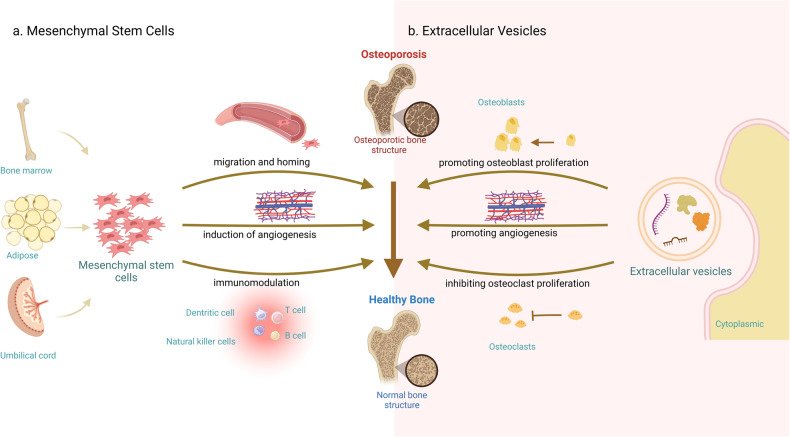
Mechanisms of OP treatment using MSCs and EVs. **a** MSCs, derived from diverse tissue origins, primarily address OP through targeted homing, angiogenesis stimulation, and immunomodulatory actions. **b** The therapeutic impact of secreted EVs is predominantly achieved by fostering osteoblast proliferation, impeding osteoclast propagation, and augmenting angiogenesis. Mesenchymal stem cells and extracellular vesicles of different origins have a positive effect on the restoration of bone density. Generated by BioRender.

MSCs represent a highly abundant class of adult stem cells that are widely investigated across the world. MSCs possess self-renewal and multilineage differentiation capabilities, enabling their differentiation into various cell types, such as adipocytes, osteoblasts, and chondrocytes [14], thereby playing an instrumental role in sustaining the equilibrium of bone physiology. MSCs can also suppress the proliferation and function of several major immune cells, including T and B lymphocytes, dendritic cells, and natural killer cells, thereby modulating immune responses [15, 16]. MSCs primarily employ three mechanisms to treat OP: 1. Migration and homing, whereby MSCs, upon receiving specific signals, migrate to the site of injury to exert their tissue repair effects [17]. 2. Induction of angiogenesis, as numerous preclinical and clinical studies have demonstrated that MSCs promote angiogenesis through vascular endothelial growth factor (VEGF), hepatocyte growth factor (HGF), Fibroblast Growth Factor 2 (FGF2), and angiogenin [18]. 3. Immune modulation, where one mechanism by which MSCs exert immunomodulatory effects is through monocyte phagocytosis of injected MSCs, thereby stimulating and inducing immune responses [19] (Fig. 1).

Nevertheless, the enduring propagation of MSCs poses a heightened possibility of tumorigenesis, attributed to the dysregulation of genes associated with the cell cycle and increased chromosomal instability [20]. Simultaneously, challenges such as cellular dedifferentiation and immunological rejection further hinder the clinical implementation of MSCs [21]. Contemporary research has uncovered that MSCs predominantly exert their impact on diseases via the release of EVs [22]. In contrast to MSCs, EVs possess superior attributes, including immunological quiescence, non-carcinogenic nature, enhanced stability, cell and tissue-specific targeting, and lack of vascular obstruction [23]. Two main subtypes of EVs exist—exosomes and ectosomes [24]. Exosomes are small (approximately 50–150 nm in diameter) vesicles formed by the inward budding of the endosomal membrane to create intraluminal vesicles (ILVs); these ILVs are secreted as exosomes when multivesicular bodies (MVBs) fuse with the plasma membrane [25]. Ectosomes, which range in size from less than 100 nm to several micrometers in diameter, encompass microvesicles, microparticles, and large vesicles. Ectosomes are formed through the outward protrusion and subsequent shedding of the plasma membrane into the extracellular space [26]. Throughout the orchestrated progression of programmed cell demise, apoptotic bodies originating from cellular remnants are likewise classified as EVs. In previous investigations, EVs have often been categorized according to exosomes, microvesicles, and apoptotic bodies. In the ensuing discourse, the narrative will unfold based on specific research studies. Predominantly, EVs modulate the functionality of recipient cells via three discrete mechanisms: (1) the interaction of transmembrane proteins present on the EVs membrane with corresponding receptors on the cellular membrane, consequently initiating signaling cascades that impact target cells [27]; (2) the amalgamation of EVs with the cell membrane, facilitating the conveyance of bioactive constituents into the cytoplasm, thereby modulating or altering intracellular signaling pathways; (3) the internalization of EVs into cells through endocytosis, culminating in the release of their cargo into designated organelles [28]. Upon fusion, mRNA transferred via EVs can be translated into proteins, while conveyed microRNAs (miRNAs) regulate mRNA translation and participate in various biological processes [29], including promoting osteogenesis, bone regeneration, and mineralization, as well as vascular network formation [30]. In contemporary research, EVs have emerged as crucial mediators of intercellular communication, given their capacity to ferry not only membrane proteins and lipids, but also RNA, cytoplasmic proteins, and a variety of signaling molecules to the receiving cells. EVs can paracrine-influence cell phenotypes, recruitment, proliferation, and differentiation. EVs functions primarily depend on their cargo, exhibiting diverse functionalities when laden with distinct materials, such as the accumulation of age-related molecules within the bone microenvironment potentially leading to OP [31], or serving as vehicles for relevant drug treatments [32]. EVs’ therapeutic effects on OP mainly manifest through promoting angiogenesis, modulating immune responses and inflammation [33], stimulating osteoblast proliferation and differentiation, and inhibiting osteoclast proliferation and differentiation [34] (Fig. 1).

## Overview of MSCs and derived EVs in the treatment of OP

### Overview of MSCs in the treatment of OP

**Fig. 2:**
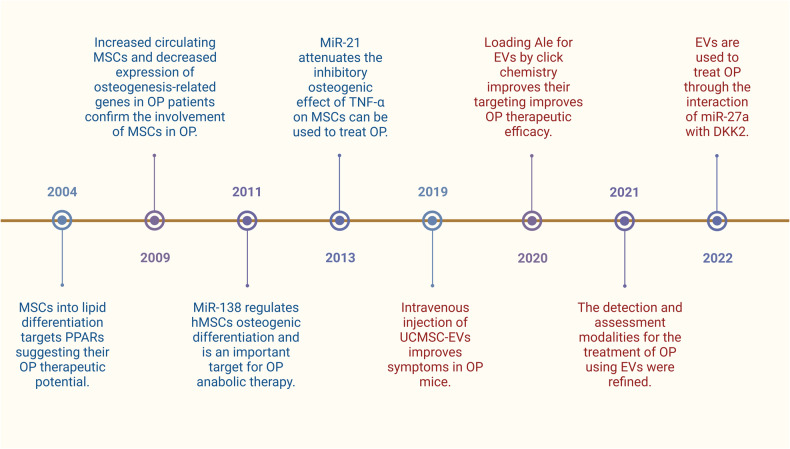
A review of previous articles using MSCs and EVs to treat OP based on LCS scores. Employing MSCs and EVs for addressing OP commenced early on, with MSCs being utilized for OP treatment prior to EVs. Numerous investigations have delved into the molecular underpinnings of these therapeutic approaches, and a multitude of preclinical trials have been conducted for validation purposes. Presently, a growing body of research is adopting engineering techniques to enhance the efficacy of these treatments. Generated by BioRender.

We employed the Histcite analysis tool to conduct a statistical analysis of literature related to MSCs and OP within the Web of Science database. Key studies were selected for review based on their Local Citation Score (LCS) rating. Ever since Rodríguez’s seminal publication in 2000, which established a link between OP and MSCs [35], there has been a significant surge in research exploring the pathogenesis and therapeutic potential of MSCs in the context of OP. In a groundbreaking study conducted in 2004, Nuttall ME and Gimble JM delved into the regulatory mechanisms governing MSC differentiation into osteoblasts or adipocytes, employing gene silencing and overexpression techniques. Their findings revealed that the activation of peroxisome proliferator-activated receptors (PPARs) stimulates adipogenesis while simultaneously suppressing osteogenesis, thereby unveiling promising targets for the development of future OP treatments [36]. In 2006, Gimble JM published a review questioning the inverse relationship hypothesis between adipocytes and osteoblasts within the bone marrow cavity, focusing on the mechanisms of MSCs differentiation and emphasizing the potential of alternative therapies for treating OP [37]. In a 2009 scholarly investigation, elevated levels of circulating MSCs were identified in OP patients via in vitro assays. This observation was accompanied by a diminished expression of Runt-related transcription factor 2 (Runx2), Sp7, collagen type I alpha 1 chain gene (COL1A1), secreted protein acidic and rich in cysteine (SPARC), and SPP1 genes, further substantiating the alterations in osteoblast differentiation that could potentially be associated with the etiology of OP [38]. In 2010, studies began to delve deeper into the upstream pathways regulating bone homeostasis by MSCs, discovering miR-204/211 as an essential endogenous negative regulator of Runx2, capable of inhibiting the osteogenesis of mesenchymal progenitor cells and bone marrow-derived mesenchymal stem cells (BMMSCs) while promoting adipogenesis [39]. In 2011, an investigation employed miR-138 antagonists in vitro analyses to substantiate that the suppression of miR-138 function by anti-miR-138 facilitates the upregulation of osteoblast-specific genes, alkaline phosphatase (ALP) activity, and matrix mineralization. This implies that the pharmacological targeting of miR-138 through anti-miR-138 inhibition could serve as a potential therapeutic approach for augmenting bone formation in vivo [40]. In 2013, the research uncovered a link between osteogenesis-regulating miRNAs and tumor necrosis factor-alpha (TNF-α), indicating a molecular basis for novel treatment strategies targeting OP and other inflammatory bone diseases [41]. A 2016 review summarized various upstream pathways and molecular mechanisms regulating the differentiation of MSCs, holding significant value for better clinical application of MSCs in tissue engineering and regenerative medicine [42] (Fig. 2).

### Overview of MSCs derived EVs in the treatment of OP

A comprehensive examination of seminal publications in recent years concerning the management of OP utilizing EVs derived from MSCs, as assessed by LCS rankings. In 2019, Hu’s study revealed that intravenous administration of umbilical cord mesenchymal stem cell derived extracellular vesicles (UCMSC-EVs) led to improvements in age-induced osteoporotic symptoms in mice, evidenced by increased trabecular and cortical bone mass. The osteogenic effect of UCMSC-EVs on BMMSCs was verified to be mediated by the highly enriched miR-3960 within them, using specific miRNA inhibitors [43]. Despite the absence of tissue-specific targeting in EVs, alendronate possesses the unique ability to selectively target bone tissue via hydroxyapatite interaction. Yayu Wang ingeniously amalgamated EVs with alendronate, yielding Ale-EVs through the application of “click chemistry,” which exhibited favorable therapeutic outcomes in an ovariectomy-induced osteoporotic rat model [44]. Utilizing sophisticated methods such as micro-positron emission tomography (μPET)/computed tomography (CT), μCT, and optical imaging, Cheng-Hsiu Lu meticulously examined osteoblastic activity, microstructure, and in vivo dynamics of EVs. Additionally, RNA sequencing was employed to scrutinize their cargo and therapeutic influence on bone tissue in ovariectomized mice, offering a comprehensive assessment of EVs’ efficacy in addressing OP and delving into molecular targets pertinent to bone remodeling regulation [45]. Yan Wang discovered the therapeutic effects of EVs on OP through the interaction between miR-27a and DKK2 by modulating the expression of miR-27a and validating the relationship between miR-27a and DKK2 [46] (Fig. 2).

In comparison with stem cells, recent years have witnessed a notable increase in the exploration of EV-based therapies for OP through previous research. However, the utilization of EVs in clinical treatment still encounters substantial challenges due to limitations in EVs extraction techniques and the inherent instability of natural EVs. Technological breakthroughs have emerged in recent years, showcasing the significant efficacy of EVs in the treatment of various diseases. This has led to advancements in the more efficient extraction and identification of EVs derived from cellular sources. Common methods for extracting EVs include ultracentrifugation and polymeric precipitation; however, both methods have their drawbacks. For instance, although ultracentrifugation is straightforward, it suffers from low yield, while precipitation methods, despite their high efficiency, are associated with lower purity [47, 48]. In recent times, scientists have successfully engineered a microfluidic chip capable of recognizing specific surface antibodies. This chip facilitates the efficient collection of targeted EVs while ensuring their purity, thereby providing technological support for expanding research in EV-related studies [49, 50]. In addition to advancements in extraction techniques, researchers are actively exploring alternative methods to enhance EVs yield and properties. This is primarily achieved by subjecting parent cells, specifically MSCs, to various stimuli to facilitate the secretion of EVs. For instance, the application of appropriate mechanical stimulation has been demonstrated to boost the secretion of osteogenesis-related EVs [51], while activation of adipose-derived mesenchymal stem cells (ADSCs) by pro-inflammatory factors such as interferon-γ (IFNγ) and TNFα can exert immunosuppressive effects [52].

**Fig. 3:**
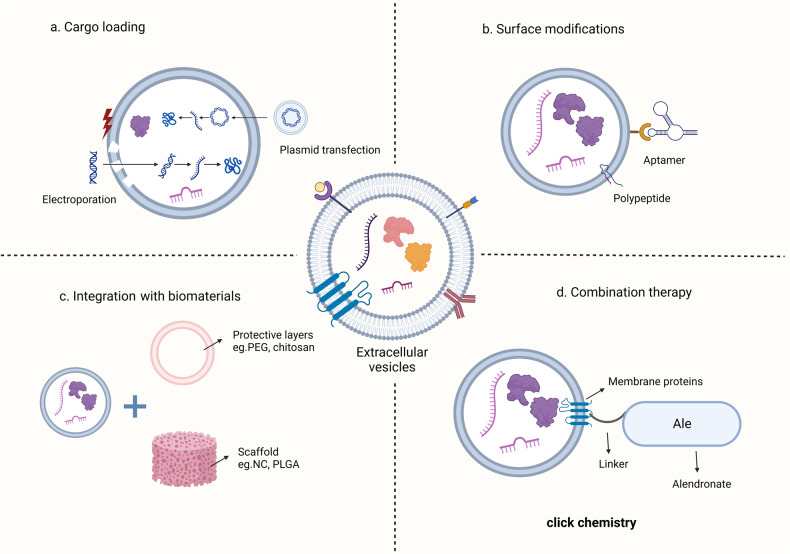
Engineering strategies in the use of EVs for the treatment of OP. **a** Cargo loading, by modulating the expression of cargos within EVs through plasmids or electroporation to act as a therapeutic OP. **b** Surface modification, by adding aptamers or specific peptides to the surface of EVs it is possible to specifically target EVs to target cells and improve the efficiency of EVs therapy. **c** Combined biomaterials, by applying a protective layer to the surface of EVs or loading EVs on a biological scaffold it is possible to reduce the loss of delivered EVs, control the rate of EVs release in the release rate in vivo and improve the stability of EVs. **d** Combination therapy, by combining EVs with therapeutic drugs through click chemistry to amplify the therapeutic effect. Generated by BioRender.

In addition to the advancements in extraction techniques, the engineering modification of EVs has significantly optimized their therapeutic efficacy. Numerous studies have confirmed that engineered EVs exhibit enhanced therapeutic potential [53]. Primary means of engineering EVs include loading therapeutic cargo within, surface modification, and amalgamating materials, as well as combination therapies. Cargo loading chiefly involves incorporating biologically active molecules with therapeutic properties, such as proteins, small molecules, or nucleic acids (e.g. miRNA, siRNA), into EVs through electroporation [54], plasmid transfection [55], or incubation with permeabilizing agents [56]. Some studies have also encapsulated RNA within EVs by generating lipid-coated RNA particles and integrating them into purified EVs via mixing-induced distribution [57]. However, this process slightly enlarges the size of the EVs, and whether this impacts their functionality warrants further investigation. With the maturation of clustered regularly interspaced short palindromic repeat (CRISPR)-associated protein 9 (Cas9) technology, the development of genetically engineered vesicles for disease treatment has progressed significantly. Engineered EVs capable of delivering specific miRNAs or small interfering RNAs (siRNAs) have been developed for the treatment of central nervous system disorders and cancers [58, 59]. Surface modification entails attaching targeting ligands, such as peptides or antibodies, to the EV surface, enabling their specific interaction with receptors expressed on the surface of target cells (e.g. osteoblasts and osteoclasts), thereby mitigating potential off-target effects [60]. For treating OP through EVs, common surface modification approaches involve the incorporation of bone-targeting ligands or bone-targeting G protein-coupled receptors onto the cell membrane, such as C-X-C motif chemokine receptor 4 (CXCR4) [61]. Incorporating materials can be achieved not only through the conventional association with scaffold materials but also by employing membrane engineering to introduce protective coatings on the vesicle surface. While numerous studies have affirmed the therapeutic efficacy of directly infusing EVs into target sites, the limited local diffusion capacity of natural EVs results in a short duration of action. To mitigate the risk of infection and reduce the frequency of EVs infusion, various types of biomaterials have been utilized in the development of scaffold systems for EVs delivery [62]. Beyond providing structural support, these biomaterials have been demonstrated to offer additional auxiliary signals, facilitating osteogenesis [63, 64]. Membrane engineering refers to the incorporation of substances such as polyethylene glycol, chitosan, or alginate salts into EVs membranes to enhance their stability during circulation, prolong their half-life, and facilitate more effective delivery to target tissues [65]. While loading therapeutic cargo onto EVs can enhance their efficacy for disease treatment, direct administration often leads to off-target side effects [66]. Consequently, many studies concurrently perform targeted modifications on the membrane of EVs when loading therapeutic cargo, representing a form of combinatorial therapy. Combining therapies refers to the integration of various engineered strategies or the conjunction of specific technical approaches with traditional treatment methods. In addition to commonly employing vesicles as carriers for drug transport, there is the utilization of click chemistry techniques to combine vesicles with drugs for the treatment of OP [44]. Alternatively, a sequential application with other treatment modalities is explored to amplify therapeutic outcomes. The ensuing discussion will primarily focus on the direct transplantation for preclinical research, exploration of molecular mechanisms, and the application of engineering strategies in the treatment of OP with EVs. The discussion will no longer redundantly delve into the individual consideration of various engineered therapeutic approaches (Fig. 3).

## Application of different MSCs-derived EVs in the treatment of OP

### Treatment of OP by BMMSCs-derived EVs

#### Molecular mechanism of BMMSC-EVs for the treatment of OP

Numerous studies have demonstrated that the direct transplantation of EVs derived from MSCs can effectively alleviate various symptoms of OP, with most investigations delving into the molecular mechanisms of utilizing EVs for OP treatment. Zuo’s research discovered that the transplantation of exosomes derived from BMMSCs mitigates bone loss in radiation-induced rat models, exhibiting a reduction in oxidative stress, accelerated DNA damage repair, and restoring the balance between adipogenic and osteogenic differentiation via the promotion of β-catenin expression in BMMSCs [67]. P Zhao co-cultured BMMSC-Exos with the osteoblastic cell line of human osteoblast-like cells (hFOB 1.19), finding that BMMSC-Exos promote the proliferation of hFOB 1.19 cells. Further research confirmed that BMMSC-Exos improve OP by stimulating osteoblast proliferation through the MAPK pathway [68]. Li’s investigation revealed that EVs derived from BMMSCs can augment osteogenesis in ovariectomized (OVX) OP rats by transferring miR-186 via the Hippo signaling pathway [69]. Current research on EVs predominantly focuses on exosome subtypes, with limited attention given to other EVs. For example, studies on apoptotic bodies for treating OP suggest that phagocytosis of apoptotic cells may induce molecular memory in macrophages, thus speculating that apoptotic bodies could facilitate intercellular communication via cytokine transfer [70]. Dawei Liu and colleagues found that systemic infusion of apoptotic bodies derived from murine BMMSCs salvaged MSC damage, directly stimulated bone formation, indirectly suppressed osteoclast activity, and ameliorated the reduced bone mass phenotype in OVX mice [71]. Yuan Zhu delved further into the role of apoptotic bodies in BMMSCs during osteogenesis, discovering that apoptotic bodies mitigate bone loss induced by primary and secondary OP while stimulating bone regeneration in defect areas. Apoptotic bodies promote osteogenesis in target cells by releasing miR1324, which suppresses the expression of target gene sorting nexin 14 and consequently activates the SMAD1/5 pathway [72]. Sylvia Weilner and colleagues revealed that senescent endothelial cells secrete microvesicles containing miR-31, which inhibit the osteogenic differentiation of MSCs [73] (Table 1).

**Table 1:** Molecular mechanism of MSCs-derived EVs in the treatment of OP.

Source of EVs	Terminology	Cargo and modifications	Model	Effect	Ref.
BMMSCs	Exosomes	Natural	Radiation-induced rat models	Promote the expression of β-catenin in BMMSCs	[67]
	Exosomes	Natural	OVX mice	Improve OP by promoting the proliferation of osteoblasts	[68]
	Exosomes	MiR-186	OVX rats	Promote osteogenesis	[69]
	Apoptotic bodies	Not revealed	OVX mice	Ameliorate the reduced bone mass phenotype	[71]
	Apoptotic bodies	MiR-1324	OVX mice	Stimulate bone regeneration in defect areas	[72]
	Microvesicles	MiR-31	In vitro	Inhibit the osteogenic differentiation of MSCs	[73]
ADSCs	EVs	MiR-21-5p	OVX mice	Inhibit osteoclast differentiation and reduce gene expression associated with bone resorption	[92]
	Exosomes	No application	In vitro	Antagonize H/SD induced osteocyte apoptosis and osteocyte-mediated osteoclastogenesis	[93]
	Exosomes	No application	Diabetic OP models	Suppress NLRP3 inflammasome activation in osteoclasts, and reduce bone resorption	[94]
	Exosomes	Not revealed	GIOP rats	Alleviate apoptosis and oxidative stress and prevent the development of GIOP	[95]
UCMSCs	EVs	Not revealed	HLU-induced DOP rat models	Prevent bone loss and maintaining bone strength	[101]
	Exosomes	Not revealed	OVX mice	Promote BMMSC proliferation and osteogenesis through the AKT signaling pathway	[102]

#### Engineering modifications of BMMSCs-derived EVs

By modifying the contents of EVs, the therapeutic efficacy of EVs for OP can be augmented. EVs contain an abundance of cargo, including hundreds to thousands of distinct proteins, unique lipids, specific DNA, and numerous small non-coding RNAs, serving as unparalleled information conveyors between cells [74]. Among the diverse cargoes carried by exosomes, small RNAs, particularly miRNAs, play a pivotal role in cellular communication [75]. Xu and colleagues documented that an increase in miR-31a-5p levels within exosomes derived from rat BMMSCs facilitates osteoclastogenesis, which consequently contributes to age-associated bone deterioration. Conversely, incorporating antagomiR-31a-5p into the bone marrow milieu attenuates osteoclast function, thereby presenting a promising therapeutic avenue for addressing OP [76]. Murong You transfected BMMSCs with miR-21-5p mimics or inhibitors to overexpress or knock down miR-21-5p, demonstrating that BMMSCs-derived exosomes with upregulated miR-21-5p further enhanced the proliferative effects on hFOB1.19 cells, while those with downregulated miR-21-5p attenuated these cellular phenotypes. The findings suggest that BMMSCs-derived exosomal miR-21-5p ameliorates OP by modulating KLF3 [77]. Min Qiu injected BMMSC-Exos transfected with miR-150-3p into OVX rat models, discovering that elevated miR-150-3p levels enhance osteoblast apoptosis, providing novel insights for the treatment of OP patients [78]. Long non-coding RNAs (lncRNAs) have emerged as promising novel modulators in the osteogenic process of MSCs. Yang’s research discovered that lncRNA MALAT1 promotes the expression of SATB2 by interacting with miR-34c, while enhanced SATB2 has been demonstrated to foster osteogenic differentiation in BMMSCs of patients with osteonecrosis [79]. In addition to nucleic acids, other cargo has also been employed to enhance vesicular efficacy. Existing research has reported that Glycoprotein Non-Metastatic Melanoma Protein B (GPNMB) is a multifunctional transmembrane glycoprotein that plays a crucial role in rescuing the decline of BMMSCs osteogenic differentiation induced by dexamethasone (Dex) [80]. Ba Huang transfected lentiviral vectors overexpressing GPNMB into BMMSCs, extracted GPNMB-EVs from the conditioned medium of GPNMB-modified BMMSCs, and found that GPNMB-EVs can stimulate BMMSC osteogenesis by activating the Wnt/β-catenin signaling pathway, indicating the vast potential of GPNMB-EVs as a cell-free therapy for treating OP [81].

Aptamers are frequently employed as tools for surface modification of EVs. As single-stranded DNA/RNA oligonucleotides, aptamers possess the ability to bind target molecules with high affinity and specificity through their three-dimensional structures [82]. Consequently, researchers have constructed aptamer-functionalized bone BMMSCs-derived exosomes (BMMSC-exo-apt) by integrating BMMSC-specific aptamers with BMMSCs-derived exosomes. Luo implemented modifications to the 5’-UTR region of the aptamer by incorporating an aldehyde moiety, which subsequently underwent a reaction with amino groups present on the exosome membrane proteins. This process led to the formation of robust Schiff base linkages, thereby considerably enhancing the in vitro uptake of BMMSC-Exos by BMMSCs. When the resultant BMMSC-Exo-Apt conjugate was intravenously administered to OVX mice, an experimental model of OP induction, there was a notable increase in bone mass and expedited bone regeneration observed in femoral fracture paradigms [83].

In integrating EVs with materials, the prevalent approach involves the combined use of EVs and various natured biocompatible scaffold materials for disease therapeutics. Xie’s research discovered that EVs derived from BMMSCs promote bone formation when combined with a demineralized bone matrix scaffold [84]. The utilization of bioactive glass nanoparticles (BGNs) in the regeneration of bone tissue has attracted considerable interest owing to their distinctive osteogenic capabilities. In vivo research has demonstrated that the combination of BGNs and BMMSCs-EVs effectively counteracts bone deterioration in osteoporotic mice, restores biomechanical characteristics of the murine femur, enhances peripheral blood biochemical markers associated with bone metabolism, and displays negligible acute systemic toxicity [85]. Anqi Liu developed a lyophilized delivery system of BMMSC-OI-exo (osteogenically induced BMMSC-Exos) on multilevel mesoporous bioactive glass (MBG) scaffolds, achieving bioactivity maintenance and sustained release by entrapping exosomes within the scaffold’s micropores. This approach effectively enhanced the scaffold’s osteogenic capacity while expediting the initiation of bone regeneration [86]. Irfan Qayoom employed a nanocement (NC) base composed of calcium sulfate/nano-hydroxyapatite as a carrier for recombinant human bone morphogenetic protein-2, zoledronic acid (ZA), and BMMSC-Exos. The results indicated that NC serves as an effective carrier for bioactive molecules, reducing the risk of hip fractures in osteoporotic animals [87] (Table 2).

**Table 2:** Engineering modifications of different MSCs-derived EVs in the treatment of OP.

Source of EVs	Terminology	Engineering Tools	Cargo and modifications	Model	Effect	Ref.
BMMSCs	Exosomes	Genetic modifications	AntagomiR-31a-5p	Age-related osteoporotic mouse models	Reduce osteoclast activity	[76]
	Exosomes	Co-cultivation, gene engineering	MiR-21-5p inhibitor	In vitro	Improve OP	[77]
	Exosomes	Genetic modifications	MiR-150-3p	OVX rats	Promote osteoblast proliferation and differentiation	[78]
	Exosomes	Co-cultivation, gene engineering	LncRNAMALAT1	OVX mice	Promote osteogenic differentiation of BMMSCs	[79]
	EVs	Content modifications	GPNMB	OVX rats	Attenuate bone loss	[81]
	Exosomes	Surface modifications	BMMSC-Exo-Apt	OVX mice	Increase bone mass and accelerate bone healing	[83]
	EVs	Integrate with biomaterials	BGN	OVX rats	Inhibit osteoclast differentiation and relieving bone loss	[85]
	Exosomes	Integrate with biomaterials, genetic modifications	MBG	Rat cranial defect model	Enhance the bone forming and induce rapid initiation of bone regeneration	[86]
	Exosomes	Integrate with biomaterials	NC	Femur neck canal defect model	Reduce the risks of fractures in osteoporotic animals	[87]
ADSCs	Exosomes	Genetic modifications	MiR-146a	Diabetic OP models	Induce the inactivation of inflammasome, and reduce bone resorption and recover bone loss	[97]
	EVs	Integrate with biomaterials	Bio-Ppy-Ti	In vitro	Exhibit superior cellular compatibility and osteoinductive properties	[99]
	EVs	Integrate with biomaterials	PLGA	Skull defect mice	Enhance bone regeneration	[100]
UCMSCs	EVs	Genetic modifications	MiR-3960	Senile osteoporotic mice	Increase trabecular and cortical bone mass	[43]
	EVs	Genetic modifications	CLEC11A	OVX mice, HLU-induced DOP rat models	Prevent OP by maintaining bone mass and strength	[103]
	Exosomes	Co-cultivation	Hsa-mir-2110 and hsa-mir-328-3p	Estrogen-deficient OP model mice	Promote osteoblast proliferation	[104]
hiPSCs	EVs	Surface modifications, genetic modifications	SDSSD, siShn3	OVX mice	Promote osteogenesis and inhibit osteoclasts	[107]
	Exosomes	Surface modifications, genetic modifications	BT-Exo-siShn3	OVX mice	Enhance osteogenic differentiation and promote angiogenesis	[108]
USCs	EVs	Genetic modifications	ShCTHRC1, shOPG	OVX mice	Restore the reduced bone strength	[110]

### Treatment of OP by ADSCs-derived EVs

#### Molecular mechanism of ADSC-EVs for the treatment of OP

In the investigation of autologous BMMSCs transplantation for treating OP, some researchers posit that the osteogenic differentiation capacity of BMMSCs in OP patients is somewhat diminished [88]. Consequently, alternative research and therapeutic interventions utilizing ADSCs, which possess comparable osteogenic differentiation potential, have been initiated [89]. Exosomes originating from adipose-derived stem cells (ADSC-Exos) demonstrate therapeutic properties analogous to their progenitor cells due to the presence of similar bioactive compounds. These ADSC-Exos are capable of modulating immune reactions, inflammation, and fostering angiogenesis, all of which contribute to sustaining bone equilibrium [90]. Moreover, they hinder the adipogenic differentiation of ADSCs by specifically activating the Hedgehog signaling pathway [91]. Extracellular vesicles derived from ADSCs (ADSC-EVs) are not only rich in growth factors and cytokines involved in bone metabolism and MSCs migration but also effectively inhibit macrophage-driven osteoclast differentiation. Lee’s experimental research reveals that intravenous administration of ADSC-EVs counteracts bone loss in osteoporotic mice, as receptor activator of nuclear factor-κB ligand (RANKL) natural inhibitor and bone-preserving protein are highly concentrated within ADSC-EVs. Additionally, miR-21-5p and let-7b-5p present in ADSC-EVs can impede osteoclast differentiation and decrease gene expression related to bone resorption, thereby facilitating the migration of BMMSCs. As a result, ADSC-EVs represent a potential acellular therapeutic approach for OP treatment [92]. Ren’s study discovered that ADSC-derived exosomes significantly attenuated H/SD-induced MLO-Y2 cell apoptosis and osteoclastogenesis by upregulating the Bcl-4/Bax ratio, suggesting therapeutic potential in age-related bone diseases [93]. ADSC-derived exosomes also exhibit considerable potential for inflammation suppression. Lei Zhang investigated the anti-osteoporotic effects and molecular mechanisms of ADSC-derived exosomes in diabetic OP, finding that they alleviate the condition by inhibiting NLRP3 inflammasome activation in osteoclasts [94]. In recent years, due to excessive use of Dex, the incidence of glucocorticoid-induced osteoporosis (GIOP) has increased, with studies now utilizing ADSC-derived exosomes for treatment. GIOP is primarily caused by oxidative stress and mitochondrial damage. In the study conducted by Xue-wei Yao, it was discovered that exosomes derived from ADSC effectively counteract the oxidative damage induced by Dex in MC3T3-E1 cells. This is achieved through the facilitation of Nrf2 nuclear translocation and the subsequent activation of the downstream enzyme HO-1. Furthermore, these exosomes serve to diminish the accumulation of Dex-induced reactive oxygen species (ROS) and prevent the deterioration of mitochondrial membrane potential [95] (Table 1).

#### Engineering modifications of ADSCs-derived EVs

Modifying or engineering the contents of EVs can alter or enhance their therapeutic capabilities [96]. Zhang et al. procured exosomes rich in miR-146a from ADSCs overexpressing miR-146a to explore their defensive properties against osteoclast-mediated inflammation. The research outcomes indicate that ADSC-derived exosomal miR-146a efficiently attenuates the production of pro-inflammatory cytokines released by osteoclasts in response to elevated glucose concentrations, provokes the deactivation of inflammasomes, impedes bone resorption, and ultimately ameliorates bone loss in diabetic OP rat models [97].

Empirical evidence demonstrates that ADSC-EVs can enhance the biocompatibility of titanium (Ti) medical implants [98]. Chen ingeniously assembled biotinylated MSC-EVs onto biotin-doped polypyrrole titanium (Bio-Ppy-Ti) surfaces, exhibiting superior cellular compatibility and osteoinductive properties in vitro compared to pure titanium, thereby presenting promising clinical applications [99]. Li et al reported the development and assessment of a novel acellular tissue-engineered bone construct, achieved by combining ADSC-Exos with poly (lactic-co-glycolic acid) (PLGA) scaffolds, significantly augmenting bone regeneration and offering a groundbreaking therapeutic paradigm for bone tissue engineering [100] (Table 2).

### Treatment of OP by EVs derived from other MSCs

#### UCMSC-EVs

Yang et al conducted a study utilizing the application of EVs derived from UCMSC-EVs in disuse OP (DOP) rat models, which were induced by hind limb unloading (HLU). The results revealed that these EVs enhance osteogenesis, reduce bone marrow adiposity, and diminish bone resorption, ultimately contributing to the preservation of bone mass and the reinforcement of bone strength in osteoporotic rodents [101]. Ren’s investigation uncovered that human UCMSC-derived exosomes enhance osteogenesis in postmenopausal OP through the AKT signaling pathway [102] (Table 1). Hu’s research elucidated the therapeutic efficacy of UCMSC-EVs on ovariectomy-induced postmenopausal OP and tail suspension-induced DOP in murine models. Proteomic assessments indicate that EVs can facilitate the osteogenic transition of BMMSCs from adipogenic differentiation by exogenously delivering the potent osteoinductive protein CLEC11A (C-type lectin domain family 11, member A) [103]. Studies have demonstrated that pre-osteogenic induction can enhance therapeutic outcomes in exosome transplantation. Ge Yahao co-cultured varying concentrations of osteogenic cells with UCMSC-Exos and compared the treatment results in OVX mice, finding that exosomes induced by osteogenic differentiation exhibited a stronger pro-osteogenic effect, albeit with diminished capacity to promote osteoblast proliferation. The underlying mechanisms warrant further investigation [104] (Table 2).

#### hiPSC – EVs

Qi and colleagues investigated the exosomes secreted by MSCs derived from human-induced pluripotent stem cells (hiPSCs, hiPSC-MSC-Exos) in OVX rats. They discovered that hiPSC-MSC-Exos stimulated angiogenesis and bone regeneration both in vivo and in vitro, exhibiting a dose-response relationship between their efficacy and exosome concentration [105]. Zhang and associates reached similar conclusions using a rat femoral non-union model [106]. Recently, scientists developed a vesicular delivery system by combining the Ser-Asp-Ser-Ser-Asp (SDSSD) peptide with human-induced pluripotent stem cell-derived EVs, constructing bone-targeting EVs and transferring small RNA-siShn3 into them. These EVs were found to enhance the expression of slit guidance ligand 3, ultimately promoting osteogenesis, inhibiting osteoclasts, and treating OP [107]. Yongzhi Cui devised an exosome delivery system based on the secretions of MSCs originating from iPSCs-engineered exosomes BT-Exo-si Shn3. The modification of bone-targeting peptides endowed BT-Exo-siShn3 with the ability to specifically deliver siRNA to osteoblasts. The silencing of the Shn3 gene in osteoblasts enhanced osteogenic differentiation and promoted angiogenesis, achieving a multifaceted anti-osteoporotic effect [108] (Table 2).

#### USC-EVs

Genetic and epigenetic variations may impede the application of iPSCs [109], necessitating the identification of an accessible, secure, and convenient alternative stem cell source for harvesting EVs conducive to bone remodeling and regeneration. Compared to other exosomes, urinary stem cell-derived EVs (USC-EVs) can be effortlessly and limitlessly procured from human urine. Chun-Yuan Chen intravenously administered USC-EVs, obtained from healthy 28-year-old females, to OVX mice and discovered an eight-week recovery in all altered parameters induced by OVX, while three-point bending tests revealed that USC-EVs restored the reduced bone strength triggered by OVX [110] (Table 2).

## Conclusions and prospects

Cell therapies, exemplified by MSCs, and cell-free therapies, represented by EVs, have emerged as paramount subjects in the field of regenerative medicine. Some nations have already incorporated stem cell-related therapeutic products into their medical insurance coverage. As a pivotal component in the therapeutic effects exerted by MSCs, EVs overcome various limitations associated with cell therapies and traditional therapies. They not only participate in intercellular crosstalk, regulating the homeostasis of diverse microenvironments, but also demonstrate immunomodulatory and angiogenic functions. Remarkable therapeutic outcomes have been observed in clinical treatments of degenerative bone diseases such as osteoarthritis and degenerative disc disease within the orthopedic domain. According to data from www.clinicaltrials.gov as of December 2023, 92 global clinical studies have focused on EVs, with 4 reaching clinical phases 3 and 4. While clinical experiments utilizing EVs for OP are yet to be conducted, numerous studies have substantiated the outstanding therapeutic efficacy of MSC-EVs through animal and cell experiments. Among the various precursor cells in the bone microenvironment, BMMSCs have garnered the most research attention in OP treatment. Current investigations not only delve into the mechanistic role of exosomes from BMMSCs in treating OP but also explore the intricate mechanisms of apoptotic bodies and microvesicles derived from these cells in OP. Compared to BMMSCs, ADSCs offer a more facile extraction process. Additionally, they exhibit sustained osteogenic differentiation capabilities [111], rendering them a popular subject of investigation for treating OP. Additionally, EVs derived from other types of MSCs are progressively investigated for OP treatment, including UCMSCs with immunological advantages, ethically uncontentious hiPSCs, and cost-effective USCs. This article not only categorically discusses the molecular mechanisms of EVs from various sources for OP treatment but also highlights the application of engineering strategies in enhancing the therapeutic effects of these EVs. This dual focus deepens our understanding of the pathogenesis of OP and provides empirical and theoretical support for advancing clinical trials using MSC-EVs for OP.

Challenges persist in utilizing cell-derived vesicles for OP treatment. Firstly, the definition and quality control of EVs remain imperfect. While extraction and characterization techniques advance, issues persist regarding the definition of EVs and the impact of different extraction methods. Classifying EVs by size or biological origin has sparked significant debate, offering insights into their standardized application despite not being the focus of this study. EVs from different stem cell sources exhibit substantial variations, and even those from the same source may undergo physicochemical changes at different stages or following diverse treatments. Discrepancies in storage methods may also influence subsequent experiments. Thus, establishing stringent standards for the extraction, storage, and application dosage of EVs is crucial for regulating related research. Secondly, the ongoing monitoring of combination therapy techniques’ development and application is essential. While some studies have combined EV-based treatment with traditional approaches or employed various engineered strategies for OP therapy, comprehensive evaluations of the safety, efficacy duration of vesicle products, and the impact of different engineering strategies and long-term use are pending further scrutiny. Besides the discussed influence of cargo loading on vesicle physiological performance, concerns arise about potential interference with the original contents of EVs after genetic engineering or drug loading. Furthermore, assessing the compatibility between engineered materials and EVs is essential. Differentiating engineered treatments for various EVs aims to maximize therapeutic effects, constituting the primary objective of combined therapy. Lastly, a thorough comparative assessment of vesicle product sources, dosages, delivery methods, and application models is needed. Various animal models are currently employed to study OP, with different laboratories utilizing distinct vesicle sources, dosages, and administration methods. Numerous reference indicators exist for assessing treatment efficacy. Through meticulous comparison and selection, developing the most suitable EVs treatment plan tailored to different types of OP is a crucial prerequisite for future clinical translation.

With advancements in extraction and identification technologies, the physiological roles and functional mechanisms of apoptotic bodies and microvesicles are gradually being uncovered, presenting potential research avenues for understanding the pathogenesis, diagnosis, and treatment of OP. The pathogenic mechanisms of OP are intricate, varying among different types, and as research on various types of EVs deepens, the mechanisms of various vesicles in the signaling pathways leading to OP will become clearer. This is crucial for the precise development of therapeutic targets. Currently, the focus of using MSC-EVs for OP treatment is largely concentrated on small animal models such as mice and rabbits, often employing the OVX modeling approach. While these models offer vital insights for preclinical research, notable disparities exist between the pathophysiology and immunological disorders of experimental animals and human diseases. Many therapeutic effects observed in animal studies do not translate into significant results during clinical trials. Moreover, with the advancements in biomimetic materials and synthetic technologies, exosome-like nanovesicles have become a current research focal point. Optimizing vesicle performance through a synthesis of diverse engineered strategies to develop safe, reliable, and cost-effective vesicle-based products for alleviating and treating OP represents a primary research objective for the future.
